# Migrating appendicolith: A retained appendicolith causing recurrent infection and migrating to the skin

**DOI:** 10.1259/bjrcr.20210093

**Published:** 2022-03-09

**Authors:** Zachary Drew, Deepak Jain, Bhanu Mariyappa Rathnamma

**Affiliations:** 1 Department of Medical Imaging, Royal Brisbane and Women's Hospital, Brisbane, Queensland, Australia; 2 Department of Medical Imaging, Townsville University Hospital, Townsville, Queensland, Australia; 3 Department of Paediatric Surgery, Townsville University Hospital, Townsville, Queensland, Australia

## Abstract

A retained appendicolith is an uncommon complication that can arise from appendix rupture and can lead to recurrent abscess formation. We present a case of a retained appendicolith causing recurrent infection over a 12-month period in a paediatric patient. The appendicolith migrated to the left side of the abdomen and then through the abdominal wall into the subcutaneous tissues. The appendicolith was finally retrieved in a joint surgical and interventional radiology case using ultrasound guidance.

## Background

Acute appendicitis is a common paediatric surgical emergency. A retained appendicolith is a rare complication that can arise from ruptured appendicitis.^1–5^ A retained appendicolith has the potential to migrate within the body and act as a nidus for recurrent infection.^1,2^ We present a case of recurrent infection over a 12-month period occurring secondary to a migrating appendicolith.

## Clinical presentation

A 13-year-old female presented to the emergency department with a 24-h history of severe, progressive right lower quadrant pain. On clinical examination, there was tenderness at McBurney’s point. Initial bloods showed a moderate leucocytosis (white cell count 15.0).

## Imaging findings

**Figure 1.**
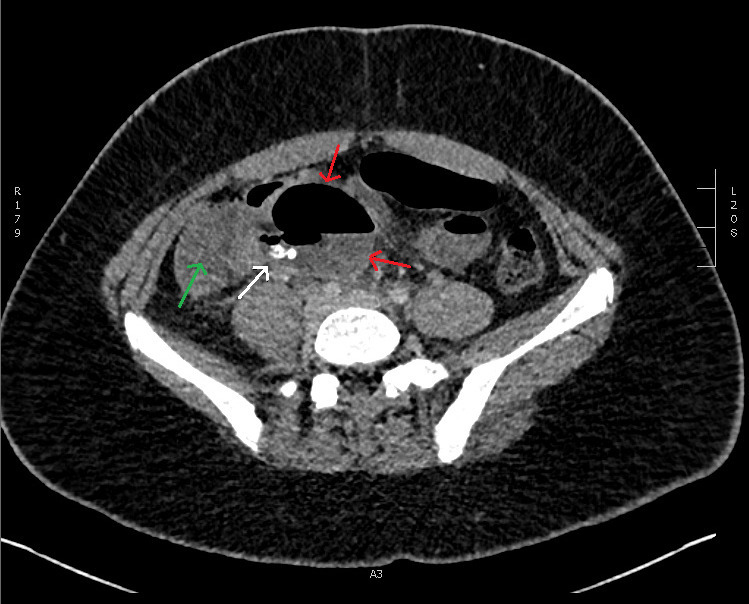
Preoperative CT (axial plane) demonstrating appendix rupture with a gas containing collection (red arrows) adjacent to the caecum (green arrow). The collection contains multiple appendicoliths (white arrow).

An ultrasound scan was performed that demonstrated a collection within the right iliac fossa. A contrast-enhanced computed tomography (CT) of the abdomen/pelvis showed a right iliac fossa collection containing three small-rounded calculi, with the appendix not identifiable (Figure 1) – suggesting ruptured appendicitis with multiple appendicoliths. The patient had laparoscopic appendicectomy and drainage of the collection, and the appendicoliths were not specifically sighted. The abdomen was flushed and a drain was placed in the right iliac fossa.

**Figure 2.**
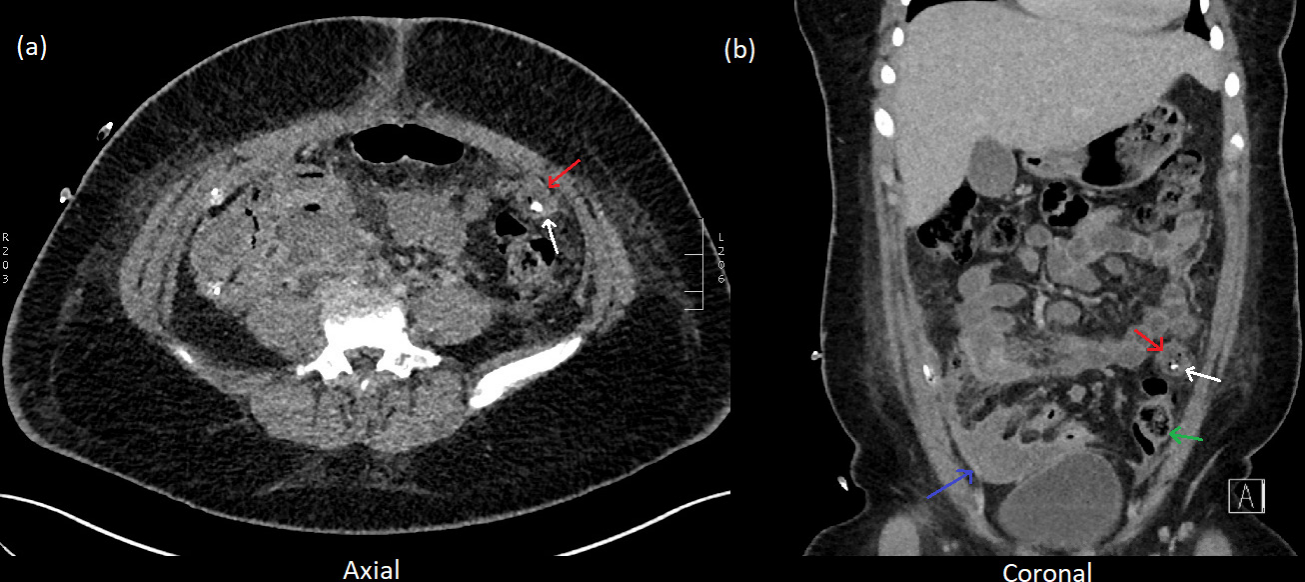
Day eight post op CT ordered for investigation of post-operative sepsis (**a**) Axial (**b**) Coronal planes. A large infected haematoma is seen within the right iliac fossa (blue arrow). A retained, migrated appendicolith (white arrow) can be seen within the left anterior abdomen with a small surrounding collection (red arrows). This was not detected at the time. The collection is noted to appear similar to closely adjacent bowel loops (*e.g.,* green arrow), which may have made detection more difficult, particularly given relative paucity of intra-abdominal fat.

The immediate post-operative course was complicated by an infected right pelvic haematoma, which required drainage and a short ICU admission. A post-operative CT (day eight post-op) demonstrating the right pelvic haematoma was retrospectively noted to show a migrated appendicolith with the left lower quadrant, which was not detected at the time. Multiple factors may have contributed to this not being identified, including the more prominent findings in the right pelvis (infected haematoma), the distance from the surgical site, the relative lack of adjacent fat stranding and the presence of a small surrounding collection mimicking adjacent bowel loops (Figure 2).

Following the drainage, the patient gradually improved and was discharged home. Two months later, however, the patient represented again with fever and pain.

**Figure 3.**
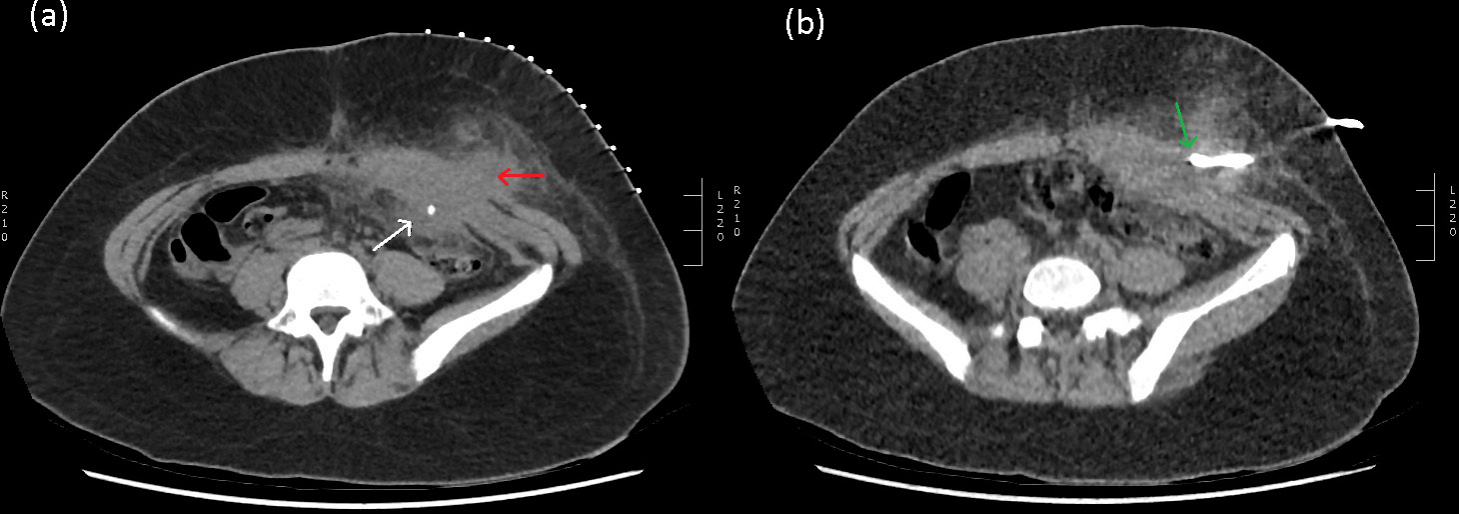
(**a**) Axial CT slice 3-month post op demonstrating an enlarged left abdominal intra-peritoneal collection extending into the subcutaneous tissues (red arrow), with the internal appendicolith seen adjacent to the abdominal wall (white arrow). (**b**) Percutaneous drainage procedure CT with the green arrow indicating the drain tip within the subcutaneous collection.

An ultrasound was performed (not shown) that demonstrated a new collection involving the subcutaneous fat overlying the left lower abdomen. As the collection extended into the superficial tissues, CT-guided percutaneous drainage was requested. The planning CT (Figure 3) confirmed a large collection in the region of the left lower abdominal surgical port tract, with an extension of the collection through the anterior abdominal wall into the subcutaneous tissue. At this time, the retained appendicolith was recognised within the intra-peritoneal aspect of the collection, having migrated from the right iliac fossa to the left anterior abdominal wall. Percutaneous drainage was performed with the calculus not retrieved. As the patient clinically improved post-drainage, the decision was made for a period of observation rather than proceed to further surgical intervention at this stage.

**Figure 4.**
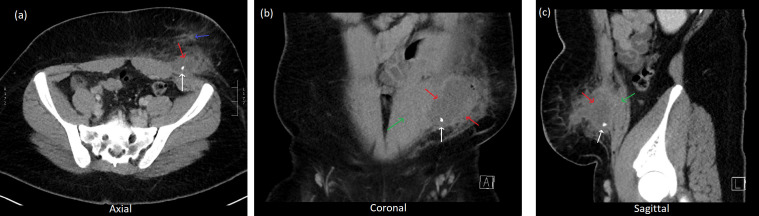
CT 11 months post op. (**a**) Axial (**b**) Coronal (**c**) Sagittal slices. There is a recurrent subcutaneous collection (red arrows) with the appendicolith (white arrows) having now migrated through the abdominal wall (just lateral to the rectus abdominal muscles (green arrow)) into the subcutaneous collection. Fat stranding is seen within the adjacent subcutaneous tissues consistent with an infected collection (blue arrows).

The patient improved and remained well up until around 11 months after her initial surgery, when she again presented with worsening left lower quadrant pain. Given the initial complications, this time a CT was performed immediately (Figure 4), which demonstrated a recurrent walled off fluid collection within the left abdominal subcutaneous tissue. This collection again contained the appendicolith, which had now eroded through the abdominal wall into the subcutaneous tissues and acted as a nidus for recurrent abscess formation.

**Figure 5.**
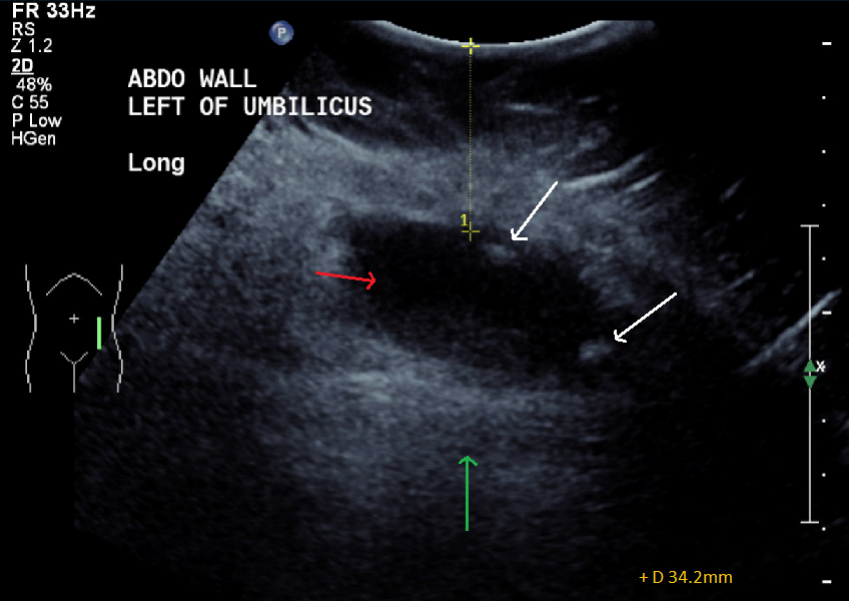
Operative planning ultrasound prior to ultrasound-guided subcutaneous collection drainage and appendicolith retrieval. The red arrow indicates the hypoechoic collection with some internal echoes overlying to the abdominal wall (green arrow), with internal appendicoliths (white arrows). The collection measured 34 mm deep to the skin.

Surgical drainage was performed, however, the appendicolith could not be found on surgery, probably due to adjacent phlegmonous changes. The collection recurred in a week and combined surgery was planned with interventional radiology in attendance, and under intra-operative ultrasound guidance (Figure 5), the calculus was identified and removed.

After the calculus was removed, the patient has remained well in the 12-month period of follow-up, with no further abdominal infections, hospital admissions or post-procedural complications to date, and was able to be discharged from the surgical clinic.

## Discussion

This case demonstrates serial abscess recurrence secondary to a migrated appendicolith post appendiceal rupture. The retained appendicolith can act as an infected foreign body which cannot be sterilised by antibiotics.^1–3^ Because of the presence of other densities of similar size within the abdomen (*e.g.,* faecoliths, phleboliths), a migrated appendicolith can be difficult to identify on conventional medical imaging. Retained appendicoliths have the potential to migrate throughout the peritoneal cavity, making identification potentially difficult. If complicated by abscess formation, the appendicoliths also have the potential to erode into other body compartments (including into the subcutaneous tissues).

A few similar case reports of migrating appendicoliths acting as a source for recurrent infection have been published, including a case of intra-thoracic migration leading to a pleural empyema (presumably from the appendicolith eroding through the diaphragm).^2^

Although uncommon, retained appendicoliths can cause significant and prolonged morbidity. This is frequently due to delayed recognition, which often occurs when there has been significant migration of the appendicolith. Increased radiologist and surgeon awareness of the potential for appendicoliths to migrate and form recurrent abscessed may help in improved detection, and potential avoidance of the associated complications, as demonstrated in this case.

## Take home messages

In conclusion, we present an uncommon case of recurrent abscess formation secondary to a retained, migrating appendicolith post-appendix rupture.

- While retained appendicoliths are a rare occurrence, it is important for the reporting radiologist to be aware of this possible complication, and review for potential retained appendicoliths particularly if there is a delayed or complicated post-operative recovery.
- Retained appendicoliths have the potential to migrate throughout the abdomen. Careful comparison with previous imaging may help in identification of new focal densities, which could potentially represent a migrated appendicolith in the setting of ruptured appendicitis.
- Retained appendicoliths usually need to be removed as they can act as a recurrent source of infection, and notifying the surgical team of this finding is imperative
